# Trauma-Associated Tinnitus and Hearing Loss: A Comprehensive Narrative Review of Prevalence, Risk Factors, and Clinical Outcomes

**DOI:** 10.3390/medicina62061164

**Published:** 2026-06-15

**Authors:** Daniel George Boicu, Oana Roxana Bitere-Popa, Romică Sebastian Cozma, Madalina-Maria Diac, Andrei Scripcaru, Cristian Marius Mârțu, Raluca Olariu, Iustin Mihai Iațentiuc, Diana Bulgaru Iliescu

**Affiliations:** “Grigore T Popa” University of Medicine and Pharmacy, 700115 Iasi, Romania; daniel.boicu2009@gmail.com (D.G.B.); sebastian.cozma@umfiasi.ro (R.S.C.); madalina-maria.diac@umfiasi.ro (M.-M.D.); andrei.scripcaru@umfiasi.ro (A.S.); martu.cristian@umfiasi.ro (C.M.M.); raluca.olariu@umfiasi.ro (R.O.); iatentiuc.iustin-mihai@email.umfiasi.ro (I.M.I.); diana.bulgaru@umfiasi.ro (D.B.I.)

**Keywords:** tinnitus, hearing loss, acoustic trauma, blast injury, traumatic brain injury, sensorineural hearing loss, early intervention, narrative review

## Abstract

*Background and Objectives*: Trauma-associated auditory dysfunction, encompassing tinnitus and hearing loss, represents a frequent yet underrecognized sequela of acoustic overexposure, blast injury, and head trauma. Despite increasing clinical awareness, the published literature exhibits substantial heterogeneity in reported prevalence estimates and recovery outcomes across different injury mechanisms. This narrative review aims to synthesize available evidence on the prevalence, clinical characteristics, recovery patterns, and prognostic factors of tinnitus and hearing loss following traumatic injury, with a particular focus on comparing outcomes across distinct trauma mechanisms and evaluating the impact of early intervention. *Materials and Methods*: A comprehensive literature search was conducted in PubMed, Embase, Scopus, and Web of Science for studies published between January 2010 and December 2025. The search strategy combined terms related to traumatic injury (e.g., “acoustic trauma,” “blast injury,” “traumatic brain injury,” “head trauma”) with terms related to auditory dysfunction (e.g., “tinnitus,” “hearing loss,” “auditory dysfunction”). Eligible studies included observational studies (cohort, cross-sectional, case–control) reporting original data on tinnitus and/or hearing loss prevalence, recovery outcomes, or prognostic factors in adult or mixed populations exposed to traumatic injury. A narrative synthesis was organized thematically around the key research questions. *Results*: The available evidence consistently indicates that tinnitus and hearing loss are frequent consequences of blast injury, acute acoustic trauma, and traumatic brain injury, although reported prevalence estimates vary considerably across studies due to differences in populations, injury mechanisms, and diagnostic criteria. Blast injury is associated with mixed hearing loss (conductive and sensorineural components), while acute acoustic trauma typically causes sensorineural hearing loss, often with a characteristic high-frequency notch. Traumatic brain injury can lead to central auditory processing deficits even when pure-tone thresholds are normal. Recovery is variable and often incomplete; tympanic membrane perforations frequently heal spontaneously, but sensorineural components often persist. Early treatment (within days to two weeks) is associated with better recovery outcomes. *Conclusions*: Trauma-associated tinnitus and hearing loss are highly prevalent and frequently result in persistent disability. The strong association between early treatment and improved recovery outcomes supports the implementation of prompt audiological evaluation and intervention following traumatic injury. These findings underscore the need for routine audiological screening in at-risk populations and for continued research into preventive strategies, standardised assessment protocols, and optimised treatment regimens.

## 1. Introduction

Traumatic injury represents a substantial and frequently underrecognized cause of auditory dysfunction. Various forms of trauma including acute acoustic trauma from intense sound exposure, blast injury from explosive overpressure waves, traumatic brain injury (TBI) from acceleration–deceleration forces or direct head impact, and whiplash-associated disorders can disrupt the structural and functional integrity of both peripheral and central auditory pathways [1,2,3]. The resulting clinical manifestations encompass a spectrum of auditory symptoms, including tinnitus, sensorineural hearing loss (SNHL), conductive hearing loss (CHL), mixed hearing loss (MHL), hyperacusis, and central auditory processing deficits [4,5].

The auditory system exhibits particular vulnerability to traumatic insults due to several anatomical and physiological characteristics. The delicate stereocilia of cochlear hair cells are susceptible to mechanical disruption from intense basilar membrane motion, while the high metabolic demands of the cochlea render it vulnerable to ischemia and oxidative stress [6,7]. Furthermore, the mammalian inner ear possesses limited regenerative capacity, as cochlear hair cells and auditory nerve fibers do not spontaneously regenerate in adult humans [8]. Consequently, trauma-induced damage to these structures often results in permanent auditory deficits.

The pathophysiological mechanisms underlying trauma-associated auditory dysfunction vary considerably by injury type. Acute acoustic trauma primarily damages outer hair cells through a combination of mechanical shearing forces and metabolic exhaustion, with the basal turn of the cochlea responsible for high-frequency perception being particularly susceptible [6,9,10]. Liberman and Kujawa [8] have demonstrated that intense acoustic exposure may also produce cochlear synaptopathy, a form of permanent neural injury affecting synapses between inner hair cells and auditory nerve fibers that can occur even when conventional audiometric thresholds recover to normal levels. This “hidden hearing loss” may explain why some individuals with a history of acoustic trauma report persistent difficulties in speech perception in noisy environments despite normal pure-tone audiometry [5,11].

Blast-related injuries involve additional mechanisms beyond those of pure acoustic trauma. The rapid overpressure wave generated by an explosion can produce tympanic membrane rupture, ossicular chain disruption, basilar membrane damage, and widespread cochlear hair cell loss [12,13]. Moreover, blast exposure frequently causes secondary TBI through rapid head acceleration, leading to diffuse axonal injury that may affect central auditory pathways including the inferior colliculus, medial geniculate body, and auditory cortex [14,15]. This combination of peripheral and central damage explains the particularly severe auditory dysfunction observed following blast injury.

Despite increasing clinical awareness over recent decades, the epidemiology and long-term outcomes of these conditions remain incompletely characterized. Reported prevalence rates of tinnitus and hearing loss following trauma vary widely across studies, with estimates ranging considerably depending on the population studied, the mechanism of injury, and the diagnostic criteria employed [16,17]. This variability reflects differences in study design, sample characteristics, assessment methods, and follow-up duration, and it complicates efforts to derive precise estimates for clinical and public health planning.

The present narrative review aims to provide a comprehensive and clinically relevant synthesis of published studies evaluating tinnitus and hearing loss following traumatic injury. The specific objectives are: (1) to describe the pathophysiological mechanisms across different trauma mechanisms; (2) to characterize the patterns of auditory dysfunction, including the distribution of SNHL, CHL, and MHL; (3) to summarize recovery outcomes following traumatic auditory injury; (4) to identify prognostic factors, particularly the impact of early versus delayed treatment initiation; and (5) to provide evidence-based recommendations for clinical practice and priorities for future research.

## 2. The Clinical and Public Health Importance of Trauma-Related Auditory Dysfunction

Auditory dysfunction following traumatic injury represents a substantial clinical and public health burden that extends far beyond the immediate post-injury period. In military populations, tinnitus and hearing loss consistently rank among the most prevalent service-connected disabilities. Epidemiological studies conducted among United States veterans have documented a high prevalence of these conditions, particularly among individuals exposed to blast-related injuries during military deployments in Iraq and Afghanistan [18,19,20]. Data from the Veterans Health Administration indicate that tinnitus and hearing loss are among the most common service-connected disabilities, affecting a large number of veterans and accounting for significant healthcare expenditures [18].

Civilian populations are also frequently affected by trauma-related auditory disorders. Motor vehicle accidents represent one of the leading causes of TBI worldwide, and auditory symptoms are commonly reported among patients with mild and moderate TBI. Oleksiak and colleagues [6] demonstrated that veterans with mild TBI frequently report tinnitus, hearing difficulties, and deficits in speech perception in noisy environments, suggesting that central auditory pathway dysfunction may play a significant role in trauma-related auditory symptoms even in the absence of peripheral hearing loss.

Blast exposure during terrorist attacks or industrial explosions can produce characteristic patterns of otologic injury. Remenschneider and colleagues [2] investigated otologic injuries following the Boston Marathon bombing and found high rates of tympanic membrane perforation, SNHL, and persistent tinnitus among survivors, highlighting the vulnerability of the auditory system to blast overpressure even in non-military contexts.

Beyond the audiological impairment itself, trauma-associated auditory disorders are associated with significant psychosocial consequences that profoundly affect quality of life. Chronic tinnitus and hearing loss frequently lead to sleep disturbance, reduced cognitive performance, anxiety, depression, social isolation, and diminished occupational functioning [21,22]. The persistent nature of these symptoms, combined with their high prevalence across diverse populations, underscores the urgent need for increased clinical awareness, early detection protocols, and effective evidence-based management strategies.

## 3. Definitions and Classifications of Trauma-Associated Auditory Dysfunction

Trauma-associated auditory dysfunction encompasses several distinct injury mechanisms characterized by different pathophysiological processes and clinical presentations. Accurate classification is essential for appropriate clinical management and prognostic counseling.

### 3.1. Acute Acoustic Trauma

Acute acoustic trauma occurs following exposure to sudden, high-intensity sound, typically exceeding 120–140 dB SPL. Such exposures may lead to mechanical disruption of cochlear hair cells, particularly outer hair cells in the basal turn of the cochlea, which are responsible for high-frequency perception [6,9]. Additionally, intense sound exposure may produce synaptic injury between inner hair cells and auditory nerve fibers a process known as cochlear synaptopathy which may lead to persistent auditory deficits including difficulties in speech perception in noisy environments even when conventional audiometric thresholds appear relatively normal [5,11]. Acute acoustic trauma is commonly observed in military personnel exposed to weapons fire, civilians exposed to explosions or loud music, and workers in noisy industrial environments.

### 3.2. Blast-Related Auditory Injury

The primary blast overpressure wave generates rapid pressure fluctuations that can produce tympanic membrane rupture, ossicular chain disruption, cochlear damage including basilar membrane rupture and hair cell loss, and various forms of vestibular injury, including vertigo/dizziness, balance or postural instability, vestibular hypofunction (unilateral or bilateral), and inner ear pathologies such as perilymph fistula or labyrinthine membrane damage [12,13]. Primary blast effects involve direct barotrauma to the tympanic membrane, ossicular chain, cochlea, and vestibular apparatus. Secondary blast effects result from penetrating injuries caused by fragmentation, while tertiary blast effects involve head trauma from acceleration–deceleration forces [2]. Blast exposure is therefore commonly associated with mixed patterns of hearing loss involving both conductive components (from tympanic membrane perforation or ossicular disruption) and sensorineural components (from cochlear hair cell damage or basilar membrane rupture). The complex and multi-level nature of blast-related auditory injury often results in particularly severe and persistent auditory dysfunction.

### 3.3. Traumatic Brain Injury and Auditory Dysfunction

TBI may impair auditory function through both peripheral and central mechanisms. Damage to the cochlea or auditory nerve may coexist with injury to brainstem or cortical auditory pathways, leading to heterogeneous auditory symptoms [14,15]. Central auditory structures including the inferior colliculus, medial geniculate body, and auditory cortex may be particularly vulnerable to diffuse axonal injury following acceleration–deceleration trauma. Gallun and colleagues [7] reported that individuals exposed to blast-related TBI frequently demonstrate deficits in auditory processing tasks involving temporal and spatial sound perception, suggesting that central auditory dysfunction may persist even when peripheral hearing thresholds recover. Some patients with TBI may experience significant auditory symptoms despite normal peripheral hearing thresholds on standard audiometric testing [20,23].

### 3.4. Whiplash-Associated Disorders

Whiplash-associated disorders, caused by rapid acceleration–deceleration movements of the cervical spine, have also been associated with auditory symptoms. Patients with whiplash injuries frequently report tinnitus, hearing disturbance, aural fullness, and dizziness [24]. Proposed mechanisms include vertebrobasilar ischemia, sympathetic nervous system dysfunction, and concurrent inner ear trauma. However, the underlying mechanisms and clinical relevance of these symptoms remain incompletely understood, and the literature on whiplash-associated auditory dysfunction remains limited [24].

## 4. Pathophysiological Mechanisms of Trauma-Related Auditory Dysfunction

The pathophysiological mechanisms underlying trauma-associated auditory dysfunction vary considerably depending on the type and severity of injury. Understanding these mechanisms is essential for developing targeted therapeutic interventions and prognostic indicators.

### 4.1. Mechanisms of Acute Acoustic Trauma

Acute acoustic trauma primarily damages outer hair cells within the organ of Corti through a combination of mechanical disruption and metabolic stress. Intense sound exposure produces excessive basilar membrane motion, leading to direct mechanical trauma to hair cell stereocilia and supporting structures [6,9]. Simultaneously, the metabolic demands of excessive stimulation may overwhelm cellular energy production, leading to oxidative stress, excitotoxicity, and apoptosis [7].

Liberman and Kujawa [8] have demonstrated that intense acoustic exposure may also produce synaptic damage between inner hair cells and auditory nerve fibers a condition now recognized as cochlear synaptopathy. This form of neural injury has been proposed as a potential mechanism underlying tinnitus and difficulties in speech perception despite relatively normal audiometric thresholds, explaining the clinical phenomenon of “hidden hearing loss” [10,11]. Importantly, cochlear synaptopathy can occur at exposure levels that do not produce permanent threshold shifts, suggesting that conventional audiometry may underestimate the true burden of acoustic injury [5].

Beyond mechanical disruption of synaptic structures, cochlear synaptopathy is driven by inflammatory and oxidative stress cascades. Acoustic overexposure triggers upregulation of pro-inflammatory cytokines, including tumor necrosis factor-alpha (TNF-α) and interleukin-1 beta (IL-1β), within the cochlea. These cytokines promote synaptic degeneration by activating nuclear factor kappa B (NF-κB) signaling in supporting cells and auditory nerve terminals, leading to further cytokine release and recruitment of reactive oxygen species (ROS)-producing cells. Simultaneously, noise-induced metabolic stress generates excessive ROS and reactive nitrogen species (RNS) in hair cells and spiral ganglion neurons, overwhelming endogenous antioxidant defenses and damaging synaptic proteins and membranes. The resulting oxidative damage, combined with inflammatory signaling, disrupts glutamate handling at the inner hair cell synapse, leading to excitotoxic swelling and retraction of auditory nerve terminals. Importantly, these inflammatory and oxidative mechanisms are potential therapeutic targets. Emerging preclinical studies suggest that anti-inflammatory agents (e.g., TNF-α inhibitors) or antioxidants (e.g., N-acetylcysteine, coenzyme Q10) administered shortly after acoustic trauma may reduce synaptopathy and preserve cochlear function [13,25]. This has direct implications for developing neuroprotective therapies for hidden hearing loss following acute acoustic trauma and blast injury.

### 4.2. Mechanisms of Blast-Related Injury

Blast-related injuries involve multiple concurrent mechanisms that distinguish them from pure acoustic trauma. The primary blast overpressure wave generates rapid pressure fluctuations that can produce tympanic membrane rupture, ossicular chain disruption, cochlear damage including basilar membrane rupture and hair cell loss, and vestibular injury [12,13]. Patterson and Hamernik [9] demonstrated that blast overpressure induces structural and functional changes throughout the auditory system, with the extent of injury depending on peak overpressure, duration, and number of exposures.

Additionally, blast exposure may produce secondary TBI through rapid acceleration of the head, leading to diffuse axonal injury affecting central auditory pathways [14,15]. This combination of peripheral and central damage explains the particularly high prevalence and severity of auditory dysfunction following blast exposure. Experimental studies have shown that blast-induced auditory injury involves not only mechanical disruption but also inflammatory and apoptotic pathways that may continue to evolve hours to days after the initial insult [13,25].

The amplification effect arises because diffuse axonal injury (DAI) preferentially disrupts the highly branched, metabolically demanding auditory pathways from the cochlear nucleus to the auditory cortex. DAI impairs temporal integration, binaural processing, and top-down modulation functions that normally compensate for peripheral cochlear damage. Consequently, a given degree of peripheral hair cell loss or cochlear synaptopathy produces a more severe functional deficit when central pathways are also injured. This synergistic interaction explains why blast-exposed individuals often report persistent tinnitus, sound localization deficits, and marked difficulty understanding speech in noise, even after peripheral thresholds have partially recovered. Experimental models confirm that combined peripheral and central injury yields disproportionately greater auditory dysfunction than either insult alone.

### 4.3. Mechanisms of Auditory Dysfunction in Traumatic Brain Injury

In TBI, auditory dysfunction may arise from damage at several levels of the auditory pathway. Peripheral injury to the cochlea or auditory nerve may occur concurrently with head trauma, particularly when temporal bone fractures are present [14,15]. However, even in the absence of peripheral damage, brainstem or cortical injury affecting auditory processing networks may produce significant auditory symptoms.

The diffuse axonal injury characteristic of acceleration–deceleration trauma may disrupt the complex neural networks required for sound localization, temporal processing, and speech perception in competing noise [23]. This explains why some TBI patients experience substantial auditory disability despite normal audiometric thresholds. Neuroimaging studies have demonstrated alterations in white matter integrity within auditory processing regions following TBI, providing a structural correlate for these functional deficits [26].

It is essential to distinguish between brainstem and cortical auditory pathway injuries in TBI, as they produce distinct clinical phenotypes and require different rehabilitation approaches.

Brainstem auditory pathway injury (affecting the inferior colliculus, medial geniculate body, or their connecting tracts) typically presents with impaired sound localization, reduced ability to extract temporal cues, and abnormal auditory brainstem response (ABR) waveforms despite normal pure-tone thresholds. Patients often report difficulty following rapid speech or localizing sounds in space. These deficits reflect disruption of early, subcortical processing stages that are critical for binaural integration and temporal precision. Rehabilitation for brainstem injuries focuses on bottom-up auditory training, including temporal processing exercises, interaural timing difference tasks, and the use of assistive devices that enhance binaural cues (e.g., frequency modulation systems).

Auditory cortex injury (affecting Heschl’s gyrus, planum temporale, or associated associative areas) produces a different phenotype: patients typically have normal ABR and sound localization abilities but exhibit deficits in higher-order processing, including difficulty understanding speech in noise, impaired recognition of non-verbal sounds (e.g., environmental sounds, prosody, music), and reduced auditory working memory. Cortical injuries may also be associated with central tinnitus, auditory agnosia, or amusia, depending on lesion laterality and extent. Rehabilitation for cortical injuries emphasizes top-down strategies: cognitive-behavioral approaches, auditory scene analysis training, metacognitive strategies for listening in noise, and environmental modifications (e.g., reducing background noise, using remote microphone systems).

Recognition of these distinct phenotypes is critical for appropriate referral: patients with suspected brainstem injury should undergo ABR and binaural processing assessment, while those with cortical injury require speech-in-noise testing and central auditory processing evaluation. Divergent rehabilitation pathways bottom-up for brainstem, top-down for cortex should be tailored accordingly, as mismatched strategies may lead to suboptimal outcomes.

## 5. Clinical Management and Outcomes

Management strategies for trauma-associated auditory disorders depend on the type, severity, and timing of injury. The evidence base for these interventions varies, and outcomes remain highly variable across patient populations.

### 5.1. Pharmacological Interventions

In cases of acute acoustic trauma or sudden SNHL, systemic or intratympanic corticosteroids are commonly administered with the aim of reducing cochlear inflammation, improving cochlear blood flow, and enhancing recovery of auditory function [27]. The clinical practice guideline for sudden hearing loss published by Chandrasekhar and colleagues [10] recommends prompt administration of corticosteroids, ideally within the first 72 h to two weeks following onset, to maximize the likelihood of hearing recovery. However, the evidence base for corticosteroid therapy specifically in trauma-related hearing loss remains limited, and treatment recommendations are often extrapolated from studies of idiopathic sudden SNHL.

This extrapolation carries important limitations. Unlike ISSNHL, which is presumed to have a predominant inflammatory or viral etiology, trauma-induced hearing loss involves mechanical disruption of cochlear structures (hair cell stereocilia, basilar membrane), intracochlear hemorrhage, and transient or permanent disruption of the blood-labyrinth barrier. These pathophysiological differences may alter corticosteroid pharmacokinetics and efficacy. For instance, hemorrhage and barrier disruption could theoretically enhance drug penetration but also increase the risk of cochlear fibrosis or reduce the bioavailability of systemically administered steroids due to impaired perfusion. No randomized controlled trial has specifically evaluated corticosteroid therapy in trauma-associated hearing loss (acoustic trauma, blast injury, or head trauma with temporal bone fracture), and current recommendations remain based on clinical experience and extrapolation from ISSNHL studies [27].

When corticosteroid therapy is considered, clinicians should weigh the routes of administration. Systemic corticosteroids (typically prednisone 1 mg/kg/day or methylprednisolone) are the conventional first-line approach, but they carry risks of hyperglycemia, hypertension, psychosis, sleep disturbance, and immunosuppression, which may be particularly problematic in polytrauma patients or those with uncontrolled diabetes. Intratympanic corticosteroids (e.g., dexamethasone or methylprednisolone injected through the tympanic membrane) offer potential advantages: higher perilymph concentrations, minimal systemic absorption, and avoidance of systemic adverse effects. Intratympanic administration is therefore an attractive option for patients with contraindications to high-dose systemic steroids (e.g., poorly controlled diabetes, active infection, severe psychiatric illness) or for those who fail to respond to systemic therapy [12,27]. However, intratympanic injection requires intact tympanic membrane access and carries small risks of persistent perforation, otitis media, or dizziness. In blast-related tympanic membrane perforation, intratympanic therapy may not be feasible acutely, and systemic administration remains the practical choice. Comparative studies between systemic and intratympanic steroids in trauma populations are lacking, and the optimal timing, dose, and duration remain undefined. Pending trauma-specific RCTs, clinical decisions should be individualized, balancing the severity of hearing loss, injury mechanism, comorbidities, and patient preferences [27].

### 5.2. Surgical Interventions

Surgical management of trauma-associated auditory injury depends on the specific structures damaged, the mechanism of injury, and the timing of presentation.

#### 5.2.1. Tympanic Membrane Perforation

Traumatic tympanic membrane perforations (TMPs) demonstrate a high rate of spontaneous healing. Observational studies report overall closure rates of 80–97% for uncomplicated traumatic perforations, with small perforations (≤30% of the tympanic membrane surface area) showing the highest probability of spontaneous closure within three to four weeks [15]. For small, uncomplicated TMPs, a watchful waiting policy for 4–8 weeks is justified, as most will close spontaneously without surgical intervention. However, larger perforations (involving >50% of the membrane surface area or with inverted edges) and those involving the margins are less likely to heal spontaneously and may benefit from earlier surgical consideration [15].

When surgical repair is indicated, tympanoplasty can be performed via either a microscopic or an endoscopic approach. Transcanal endoscopic ear surgery (TEES) has gained widespread acceptance as a minimally invasive alternative to conventional microscopic tympanoplasty. By accessing the middle ear via the external auditory canal without an external incision, TEES eliminates the need for a retroauricular or endaural incision, reduces postoperative pain, shortens operating time, avoids visible scarring, and provides superior visualization of the deep external auditory canal and anterior tympanic membrane remnant [10]. A multicenter prospective cohort study involving 13 Japanese tertiary referral centers reported that TEES was feasible in 75% of common middle ear surgeries, including 81% of chronic otitis media with tympanic membrane perforation cases and 66% of cholesteatoma cases [10].

Recent advances in endoscopic graft materials and techniques have expanded the indications for minimally invasive repair. For small-to-medium-sized perforations (≤5 mm), butterfly inlay cartilage tympanoplasty achieves comparable audiological outcomes (air–bone gap improvement 2.33–8.16 dB) to conventional underlay techniques, while being less invasive and preserving normal anatomical structures [28]. Graft uptake rates are high (89.5–100%), with significant postoperative air–bone gap improvements (9.5–10.2 dB) [11]. For subtotal perforations with minimal anterior margins, a novel composite graft (partial-thickness tragal cartilage overlaid with temporalis fascia) offers a promising alternative, achieving successful closure even in complex cases where traditional grafts may fail [11]. Additionally, the first reported endoscopic transplantation of a tympanic allograft (Allograft Tympano–Ossicular System, ATOS) for a large subtotal perforation was recently described, achieving complete closure and hearing improvement from a pure-tone average of 62 dB preoperatively to 23 dB postoperatively, without severe adverse events [8].

#### 5.2.2. Ossicular Chain Injuries

Ossicular chain disruption, which may occur in severe blast injury, penetrating trauma, or temporal bone fracture, typically requires surgical reconstruction to restore conductive hearing [2,9,29]. Traditional microscopic ossiculoplasty (OPL) remains effective, but endoscopic ossiculoplasty (endo-OPL) has emerged as a valuable alternative. A multicenter study of 292 patients undergoing endo-OPL reported significant audiological improvement, with mean preoperative air–bone gap (ABG) reducing from 26.88 dB (SD ± 12.73) to 19.94 dB (SD ± 10.90) at a mean follow-up of 20.7 months, and a graft success rate of 94.2% [12,13]. Both cement and bony partial ossicular replacement prostheses (PORP) showed consistent ABG improvements over time, with prosthesis extrusion rates of 0–8.4% [13]. A systematic review and meta-analysis comparing endoscopic versus microscopic ossicular chain reconstruction found that endoscopic ossiculoplasty had shorter operating durations and comparable audiological outcomes, with additional advantages including improved visualization, decreased need for supplemental incisions, excellent ergonomics, and reduced postoperative pain [14]. Endoscopic ear surgery is particularly valuable in traumatic ossicular injury with an intact tympanic membrane, where it enables precise diagnosis and treatment without the need for incisions or mastoidectomy [17].

#### 5.2.3. Blast Injury Considerations

Blast-related tympanic membrane perforations warrant special consideration. A study of 70 Ukrainian soldiers with persistent post-blast TMPs found that perforations involving two quadrants were associated with greater hearing loss and larger air–bone gap, and surgical intervention significantly improved hearing outcomes (*p* < 0.001) [16]. In patients with concurrent Eustachian tube dysfunction—common after blast injury—a modern combined approach involving simultaneous septoplasty, inferior turbinectomy, and tympanoplasty with prolonged middle ear ventilation has been shown to be effective, with early intervention particularly critical for faster recovery [30].

#### 5.2.4. Optimal Timing of Surgical Intervention

The optimal timing of surgical repair remains an area of ongoing debate, but current evidence supports a risk-stratified approach:

Small, uncomplicated traumatic perforations (<30% of TM area, no epithelial invagination): Watchful waiting for 4–8 weeks is justified, given spontaneous closure rates of 80–97% within three to four weeks [15]. During this period, the ear should be kept dry and water exposure avoided.

Large perforations (>50% of TM area, or involving margins): Early surgical repair (within 2–3 months) may be considered, as spontaneous closure rates are lower and persistent perforation risks middle ear infection and cholesteatoma formation [15,30].

Ossicular chain disruption: Surgical intervention can be delayed if the tympanic membrane is intact, but earlier repair (within 3–6 months) may prevent secondary middle ear fibrosis and facilitate better audiological outcomes [17]. The average interval from injury to surgery in published series ranges from several months to over five years, but hearing results remain favorable even after delayed treatment [17].

Blast injury with mixed hearing loss: Early intervention (within 2–3 months) is particularly critical for military patients to ensure faster recovery and prevent chronic middle ear complications [30].

#### 5.2.5. Impact of Surgical Timing on Hearing Outcomes

For tympanic membrane perforations, delayed repair (beyond 6 months) remains effective, but prolonged perforation increases the risk of recurrent otorrhea, middle ear infection, and cholesteatoma formation. A study on blast-related TMPs found that surgery significantly improved hearing compared to preoperative data (*p* < 0.001), with closure of the air–bone gap [16]. For ossicular chain injuries, favorable hearing results (mean ABG reduction of 24.3 dB, *p* < 0.01) have been reported even after delayed treatment, but longer delays may allow progressive middle ear fibrosis that complicates surgery [17]. Overall, the available evidence suggests that while tympanic membrane perforations can be managed conservatively initially, ossicular chain injuries benefit from timely surgical reconstruction to optimize audiological outcomes.

### 5.3. Audiological Rehabilitation

For patients with persistent hearing loss, audiological rehabilitation including hearing aids and assistive listening devices represents the primary therapeutic approach [21]. The selection of appropriate amplification devices depends on the type, degree, and configuration of hearing loss, as well as the patient’s communication needs and lifestyle factors.

Management of chronic tinnitus typically involves multimodal strategies including sound therapy, tinnitus retraining therapy, cognitive behavioral therapy, and, in some cases, pharmacological management of associated anxiety or depression [21,22,31]. Baguley and colleagues [30] emphasize that while these interventions may reduce the perceived burden of tinnitus and improve quality of life, effective curative treatments remain limited, and management focuses on habituation and coping strategies rather than elimination of the tinnitus percept.

Despite available therapeutic interventions, recovery outcomes remain highly variable, and a considerable proportion of patients develop chronic auditory sequelae. The factors predicting favorable versus poor outcomes remain incompletely understood, highlighting the need for further research in this area.

## 6. Elements of Novelty

While previous reviews have generally focused on specific injury types such as blast trauma [2,25], acoustic trauma [6,10], or TBI [15,20], the present narrative review provides a comprehensive synthesis across multiple trauma mechanisms within a unified framework.

The novelty of this review lies in several key aspects:

(a) Synthesizes current knowledge on pathophysiological mechanisms across different trauma types, allowing clinicians to understand the distinct mechanisms underlying auditory dysfunction following different forms of trauma.
(b) Enables comparison of clinical patterns across different trauma mechanisms, highlighting the particularly severe burden associated with blast injury and acoustic trauma compared to other injury types.
(c) Synthesizes available evidence on recovery outcomes and prognostic factors, emphasizing the critical importance of early intervention based on the best available evidence.
(d) Identifies persistent gaps in the current evidence base and proposes specific directions for future research, including the need for prospective longitudinal studies, standardized assessment protocols, and investigation of central auditory processing disorders.
(e) Unlike previous reviews that have focused predominantly on military populations, this review integrates evidence from both military and civilian trauma populations, enhancing these findings.

## 7. Materials and Methods

### 7.1. Study Design

This study was conducted as a narrative review of the peer-reviewed literature examining trauma-associated tinnitus and hearing loss. Unlike systematic reviews that aim to answer a narrow clinical question with quantitative synthesis, a narrative review provides a qualitative, thematic synthesis of heterogeneous evidence. This approach is particularly suited to the present topic given the diversity of trauma mechanisms (acoustic trauma, blast injury, traumatic brain injury, head trauma, whiplash), study designs (cohort, cross-sectional, case–control), outcome measures, and follow-up durations reported in the literature.

### 7.2. Research Questions

This review was guided by the following research questions, developed using the PCC (Population, Concept, Context) framework:

- **Population:** Adults and mixed populations (military and civilian) exposed to traumatic injury, including acute acoustic trauma, blast injury, traumatic brain injury (TBI), head trauma, or whiplash-associated disorders.
- **Concept:** Tinnitus and/or hearing loss (sensorineural, conductive, mixed, or central auditory processing deficits), including prevalence, clinical characteristics, recovery outcomes, prognostic factors, and the impact of early intervention.
- **Context:** Any clinical or community setting where trauma-exposed individuals are evaluated or treated (e.g., emergency departments, otolaryngology clinics, audiology centers, military treatment facilities, rehabilitation units), without geographic restriction.

### 7.3. Literature Search Strategy

#### 7.3.1. Electronic Databases

A comprehensive search was conducted in PubMed/MEDLINE, Embase, Scopus, and Web of Science Core Collection. The search was performed on 15 January 2026, and covered publications from 1 January 2010 to 31 December 2025. This time frame was chosen to capture contemporary research following the major military conflicts in Iraq and Afghanistan, which generated substantial literature on blast-related auditory injury. Although the systematic literature search was restricted to publications from January 2010 onwards, landmark studies published before 2010 (e.g., Patterson & Hamernik, 1997; Ferrari & Russell, 1999) were included manually in Table 1 because they are considered foundational to the understanding of blast-induced auditory injury and whiplash-associated auditory symptoms [9,28], respectively. These studies are cited as representative examples of the trauma types but were not retrieved by the primary electronic search due to the date restriction.

#### 7.3.2. Search Terms

The core search strategy combined terms for traumatic injury with terms for auditory dysfunction using Boolean operators (AND, OR). The detailed search string for PubMed was as follows: (“acoustic trauma”[Mesh] OR “blast injuries”[Mesh] OR “brain injuries, traumatic”[Mesh] OR “head trauma”[tiab] OR “whiplash”[Mesh]) AND (“tinnitus”[Mesh] OR “hearing loss”[Mesh] OR “hearing loss, sensorineural”[Mesh] OR “auditory diseases, central”[Mesh] OR “hidden hearing loss”[tiab] OR “cochlear synaptopathy”).

#### 7.3.3. Supplementary Strategies

Additional studies were identified through:

- **Reference list screening** (snowballing) of included studies and relevant reviews.
- **Forward citation tracking** using Google Scholar.
- **Manual searching** of key journals (e.g., Hearing Research, Ear and Hearing, Journal of Neurotrauma) for the last two years.

### 7.4. Study Selection Process

#### 7.4.1. Screening and Deduplication

All retrieved records were exported to EndNote X9 for deduplication. Two reviewers independently screened titles and abstracts against the eligibility criteria. Disagreements were resolved by discussion or by a third reviewer. Full texts of potentially eligible studies were then retrieved and assessed independently by the same two reviewers.

#### 7.4.2. Eligibility Criteria

**Inclusion criteria:**

- Observational studies (cohort, cross-sectional, case–control) or interventional studies (randomised controlled trials, quasi-experimental studies).
- Adult or mixed populations exposed to acoustic trauma, blast, TBI, head trauma, or whiplash.
- Reported original data on tinnitus prevalence, hearing loss prevalence, recovery outcomes, or prognostic factors.
- Hearing loss required objective audiometric assessment (pure-tone audiometry) or validated subjective audiometric measures (e.g., self-report questionnaires, hearing handicap inventories).
- Case series required ≥10 participants.
- Published in English in a peer-reviewed journal.

**Exclusion criteria:**

- Case reports (<10 participants).
- Animal studies or in vitro studies.
- Conference abstracts without a full manuscript.
- Reviews, editorials, commentaries (except for reference screening).
- Duplicate publications of the same population.
- Non-traumatic etiologies (e.g., presbycusis, ototoxic drugs, congenital hearing loss).

### 7.5. Data Extraction

A standardised data extraction form was developed. The form was then piloted on a random sample of five studies from the final set of included studies (after full-text screening and eligibility assessment had been completed). No major modifications were required following the pilot, and the extracted data from the pilot studies were retained in the final analysis:

1. **Study characteristics**: first author, year of publication, country, study design, setting, sample size, participant demographics.
2. **Trauma characteristics**: type of trauma (acoustic, blast, TBI, head trauma, whiplash), mechanism, severity (if reported), time since injury.
3. **Outcome measures**: definition of tinnitus, definition of hearing loss, type of hearing loss (SNHL, CHL, MHL), assessment methods (audiometry, questionnaires).
4. **Recovery outcomes**: complete recovery (return to baseline or normal thresholds), partial recovery (improvement without normalisation), no recovery. Improvement without normalisation’ refers to a statistically or clinically meaningful reduction in symptom severity (e.g., pain, tinnitus loudness) or elevation of hearing thresholds that remains outside the normal reference range or does not reach the patient’s pre-injury baseline.
5. **Prognostic factors**: timing of treatment (early vs. delayed), other predictors (age, injury severity, presence of vertigo, etc.).

Disagreements in data extraction were resolved by consensus or by a third reviewer.

### 7.6. Quality Assessment

Consistent with the methodological framework for scoping and narrative reviews, a formal risk-of-bias assessment was not performed, consistent with narrative review methodology. Instead, a qualitative critical appraisal was conducted, focusing on:

- Clarity of inclusion/exclusion criteria.
- Adequacy of sample size.
- Use of objective outcome measures.
- Completeness of follow-up (for cohort studies).
- Management of potential confounding factors.
- Disclosure of conflicts of interest.

This approach allows the reader to understand the strengths and limitations of the included evidence without producing spurious quantitative quality scores.

To minimize selection bias during the qualitative critical appraisal, we implemented several measures. First, two reviewers independently screened all titles, abstracts, and full texts against the predefined eligibility criteria; disagreements were resolved by consensus or by consultation with a third reviewer. Second, the literature search was conducted across four major electronic databases (PubMed, Embase, Scopus, Web of Science) without language restrictions beyond English, and supplementary hand-searching of reference lists was performed to identify studies potentially missed by the electronic search. Third, we explicitly documented all inclusion and exclusion decisions, including reasons for exclusion at the full-text stage, to ensure transparency and reproducibility. Fourth, we did not exclude studies based on their results or direction of effect; all studies meeting the eligibility criteria were retained for qualitative synthesis regardless of whether they reported positive, negative, or null findings. These measures, while not equivalent to formal risk-of-bias scoring, reduce the risk of selective inclusion and enhance the methodological transparency of this narrative review.

### 7.7. Synthesis of Data

Data were synthesised using descriptive summaries and thematic analysis. Studies were grouped by trauma mechanism (acoustic trauma, blast injury, TBI, head trauma, whiplash). For each group, we summarised:

- Prevalence estimates (range, not pooled).
- Patterns of hearing loss.
- Recovery outcomes.
- Prognostic factors.

Where appropriate, findings were compared across trauma types to identify commonalities and differences. No meta-analysis was performed because of the substantial heterogeneity in study designs, populations, and outcome definitions, and because the aim was to map the evidence qualitatively.

## 8. Results

### 8.1. Overview of the Included Evidence

The literature on trauma-associated tinnitus and hearing loss comprises observational studies (cohort, cross-sectional, case–control), case series (≥10 participants), clinical guidelines, and systematic reviews. Study populations include both civilians and military personnel exposed to blast injury, acute acoustic trauma, traumatic brain injury (TBI), head trauma, and whiplash-associated disorders. The evidence is heterogeneous with respect to study design, sample size, injury severity, diagnostic criteria, and follow-up duration. Table 1 provides a qualitative summary of the distribution of studies across trauma types based on the available literature.

### 8.2. Reported Prevalence Patterns

Due to substantial heterogeneity across studies in population characteristics, injury mechanisms, diagnostic criteria, and follow-up duration, no pooled prevalence estimates are provided in this narrative review. However, the literature consistently indicates that both tinnitus and hearing loss are frequent consequences of traumatic auditory injury, with prevalence estimates varying considerably depending on the specific population and injury type studied [16,17]. Table 2 summarises the reported clinical patterns for each trauma type based on the available evidence.

### 8.3. Patterns of Hearing Loss

Among studies that provide detailed audiological characterisation, sensorineural hearing loss (SNHL) is the most commonly reported type following traumatic injury, particularly after acoustic trauma and blast exposure. Conductive hearing loss (CHL) is also reported, especially in the acute phase following blast injury where tympanic membrane perforation or ossicular chain disruption may occur. Mixed hearing loss (MHL) is characteristic of blast injury, reflecting simultaneous damage to both the conductive and sensorineural structures of the ear [2,12]. Table 3 summarises the distribution of hearing loss types reported in the literature.

### 8.4. Recovery Outcomes

Recovery of auditory function following traumatic injury is variable and often incomplete. The literature reports three general patterns: complete recovery (return to baseline or normal hearing thresholds), partial recovery (improvement without normalisation), and no recovery (persistent deficit). Complete recovery is more likely in cases of isolated tympanic membrane perforation or mild acoustic trauma, while severe blast injury or acoustic trauma with cochlear hair cell loss frequently results in permanent deficits [2,6,12]. Table 4 summarises recovery patterns reported in the literature.

Distinguishing between transient and chronic auditory symptoms is essential for patient counseling and follow-up planning.

Transient symptoms typically resolve spontaneously within weeks to three months post-injury and include:

Temporary threshold shifts (TTS): Reversible elevation of hearing thresholds following acute acoustic or blast exposure, usually recovering within 24–72 h, though complete resolution may take up to 2–3 weeks. TTS results from metabolic exhaustion of hair cells without permanent structural damage.

Minor tympanic membrane perforations: Small, uncomplicated perforations (<30% of TM area) often heal spontaneously within 4–8 weeks.

Acute tinnitus post-injury: May diminish as cochlear inflammation subsides and synaptic repair occurs.

Patients with transient symptoms can be reassured about favorable prognosis, advised to avoid further noise exposure during recovery, and scheduled for follow-up audiometry at 1–3 months to confirm resolution.

Chronic deficits persist beyond 3–6 months and often represent permanent structural or functional damage:

High-frequency sensorineural hearing loss notches (most commonly at 4000–6000 Hz) following acute acoustic trauma: These are typically permanent due to irreversible outer hair cell loss in the basal turn of the cochlea.

Persistent high-frequency or tonal tinnitus following acoustic trauma or blast injury: Often remains chronic due to permanent cochlear damage and central auditory reorganization.

Central auditory processing disorders (CAPD) following TBI: Deficits in speech perception in noise, sound localization, and temporal processing may persist indefinitely, even when pure-tone thresholds normalize.

Mixed or profound hearing loss following severe blast injury or transverse temporal bone fracture: Usually permanent due to mechanical disruption of the cochlea or ossicular chain.

Patients with chronic deficits require: (i) realistic prognostic counseling about irreversibility; (ii) referral for hearing aids, assistive listening devices, or cochlear implantation as appropriate; (iii) tinnitus management strategies (sound therapy, cognitive behavioral therapy); (iv) for CAPD, specialized central auditory rehabilitation; and (v) long-term audiological follow-up (every 6–12 months) to monitor for progression or additional deficits.

### 8.5. Prognostic Factors

The timing of treatment initiation is reported as an important prognostic factor in the literature on sudden sensorineural hearing loss, and similar principles are often applied to trauma-related auditory injury. Clinical guidelines recommend prompt administration of corticosteroids, ideally within 72 h to two weeks of onset, to maximise the likelihood of hearing recovery [27]. Other reported prognostic factors include the severity of initial hearing loss, presence of vertigo, patient age, and the integrity of the tympanic membrane [2,27]. Table 5 summarises prognostic factors reported in the literature.

Pre-existing comorbidities represent an additional, often overlooked, prognostic domain. Three conditions are particularly relevant:

Diabetes mellitus is associated with microvascular injury, impaired angiogenesis, and reduced neurotrophic support, all of which compromise cochlear repair mechanisms. Hyperglycemia exacerbates noise- or blast-induced oxidative stress and inflammation, leading to more severe hair cell loss and poorer recovery of auditory thresholds. Diabetic patients also have higher rates of tympanic membrane perforation non-healing and worse post-tympanoplasty outcomes.

Hypertension contributes to cochlear damage through microvascular rarefaction, reduced cochlear blood flow, and impaired autoregulation. The inner ear is highly sensitive to ischemic injury, and hypertensive patients may experience compounded hypoxia following acoustic or blast trauma, resulting in larger permanent threshold shifts and prolonged recovery times.

Presbycusis (age-related hearing loss) reduces the baseline cochlear reserve and depletes hair cell and spiral ganglion populations. Older individuals with pre-existing high-frequency hearing loss have less neural plasticity and diminished regenerative capacity, making them more vulnerable to trauma-induced additional deficits. Consequently, the same acoustic exposure may produce more severe and persistent tinnitus and hearing loss in a presbycusic patient compared to a younger, normal-hearing individual.

Beyond patient-specific comorbidities, trauma-specific variables significantly influence the risk of permanent hearing loss and tinnitus.

Blast overpressure magnitude is a key determinant of auditory injury severity. Higher peak overpressure (typically >15–20 psi for isolated tympanic membrane rupture, with higher levels causing progressively more severe cochlear damage) correlates with greater risk of permanent sensorineural hearing loss, ossicular chain disruption, and persistent tinnitus. Experimental studies demonstrate that blast overpressure magnitude directly correlates with the extent of basilar membrane disruption, hair cell loss, and intracochlear hemorrhage. In clinical series, patients exposed to high-yield explosions (e.g., improvised explosive devices, artillery shells) consistently show worse audiological outcomes than those exposed to lower overpressure sources (e.g., small arms fire).

Exposure frequency also matters. Repeated blast or acoustic trauma exposures, even at sub-threshold levels, cumulatively deplete cochlear reserves and reduce neural plasticity. Military personnel with multiple blast deployments have a higher prevalence of clinically significant hearing loss and tinnitus compared to those with single exposures. Similarly, individuals with repeated occupational noise exposure (e.g., musicians, industrial workers) superimposed on a single acute acoustic trauma show worse recovery and higher rates of persistent symptoms. This cumulative effect reflects progressive cochlear synaptopathy and diminished regenerative capacity.

Temporal bone fracture type is a critical prognostic factor in head trauma patients. Longitudinal fractures (parallel to the petrous ridge) are more common (70–80% of temporal bone fractures) and typically spare the otic capsule. They often present with conductive hearing loss due to tympanic membrane tear or ossicular chain disruption, but sensorineural hearing loss, if present, is usually mild and may recover. Transverse fractures (perpendicular to the petrous ridge) are less common (10–20%) but carry a much worse prognosis. They frequently traverse the otic capsule, directly damaging the cochlea and vestibular apparatus. Transverse fractures are associated with profound sensorineural hearing loss (often complete and irreversible), severe vertigo, and facial nerve injury. Mixed fractures combine features of both. The distinction between fracture types on high-resolution CT is therefore essential for prognostic counseling: patients with transverse fractures should be informed of the high likelihood of permanent, severe hearing loss, whereas those with longitudinal fractures may have a better chance of hearing recovery, particularly of the sensorineural component.

### 8.6. Pathophysiological Mechanisms

The literature describes distinct pathophysiological mechanisms for different trauma types. These mechanisms explain the clinical patterns observed and are summarised in Table 6.

The synthesis of the available literature reveals several noteworthy patterns that extend beyond previously published reviews:

First, the consistent observation that mixed hearing loss is characteristic of blast injury, whereas isolated sensorineural loss predominates in acoustic trauma, underscores the importance of obtaining detailed audiological characterisation (including both air and bone conduction thresholds) in trauma patients. This distinction has direct therapeutic implications: conductive components may resolve spontaneously or require surgical intervention, while sensorineural components may benefit from early corticosteroid therapy.

Recognition that central auditory processing deficits can occur after TBI even in the presence of normal pure-tone audiometry [6,23] highlights a potential gap in current clinical practice. Standard audiometric evaluation alone may be insufficient to detect functionally significant auditory impairment in this population. This finding suggests that referral for specialised central auditory processing assessment should be considered for TBI patients who report auditory difficulties despite normal hearing thresholds.

Literature on cochlear synaptopathy [10] provides a mechanistic explanation for the clinical phenomenon of “hidden hearing loss” following acoustic trauma. This insight has important implications for medicolegal evaluation and disability assessment, as individuals with normal audiograms may still have permanent auditory injury that affects real-world communication abilities, particularly in noisy environments.

Limited evidence available for whiplash-associated auditory symptoms [24] represents a significant knowledge gap. Given the high prevalence of whiplash injuries following motor vehicle accidents, further research is needed to characterise the incidence, mechanisms, and natural history of auditory dysfunction in this population.

Consistent association between early treatment and improved recovery outcomes, although derived primarily from studies of idiopathic sudden sensorineural hearing loss [27], supports the implementation of protocols for prompt audiological evaluation and intervention following traumatic auditory injury. This finding has practical implications for emergency department and primary care settings, where auditory symptoms may be overlooked in the context of more immediately life-threatening injuries.

## 9. Discussion

### 9.1. Principal Findings and Interpretation

This narrative review synthesises evidence from a diverse range of studies examining trauma-associated tinnitus and hearing loss across different injury mechanisms. The findings consistently demonstrate that auditory dysfunction is a frequent and clinically significant consequence of blast injury, acute acoustic trauma, traumatic brain injury (TBI), head trauma, and whiplash-associated disorders. Although precise pooled prevalence estimates cannot be derived due to substantial heterogeneity across studies, the available evidence indicates that tinnitus and hearing loss occur in a considerable proportion of affected individuals, with blast injury and acute acoustic trauma showing the highest reported frequencies [1,2,3,6,7,12,16,17].

A key observation emerging from the literature is that different trauma mechanisms produce distinct patterns of auditory dysfunction. Blast injury frequently results in mixed hearing loss, reflecting simultaneous damage to the tympanic membrane, ossicular chain, and cochlea [1,2,9,12]. In contrast, acute acoustic trauma predominantly causes sensorineural hearing loss, often with a characteristic notch at 4000 Hz, due to the selective vulnerability of outer hair cells in the basal turn of the cochlea [3,6,8]. Traumatic brain injury, even in the absence of peripheral auditory damage, can lead to central auditory processing deficits that affect speech perception in noisy environments [5,6,7,16]. These mechanistic differences have important implications for clinical assessment and management.

### 9.2. Comparison with Previous Literature

The findings of this review are consistent with and extend earlier work in several ways. The observation that blast injury produces a high frequency of mixed hearing loss and persistent tinnitus aligns with the results of Remenschneider et al. [2] and Cave et al. [1], who documented substantial otologic morbidity following blast exposure, including tympanic membrane perforation, ossicular disruption, and sensorineural hearing loss. Similarly, the high prevalence of auditory symptoms following acute acoustic trauma is supported by experimental and clinical studies reviewed by Ryan et al. [3] and Liberman and Kujawa [8], who described both temporary and permanent threshold shifts as well as cochlear synaptopathy.

Recognition that TBI can cause central auditory processing deficits even when peripheral hearing thresholds are normal is consistent with the work of Oleksiak et al. [6], Gallun et al. [7], and Le et al. [5]. These studies indicate that standard pure-tone audiometry may be insufficient to capture the full spectrum of auditory impairment in TBI patients, a finding that has important implications for clinical practice and disability assessment.

Limited evidence on whiplash-associated auditory dysfunction, as noted by Ferrari and Russell [28], highlights a significant gap in the literature. Given the high incidence of whiplash injuries following motor vehicle accidents, further research is needed to characterise the nature, prevalence, and natural history of auditory symptoms in this population.

### 9.3. Pathophysiological Mechanisms and Clinical Correlates

The pathophysiological mechanisms underlying trauma-associated auditory dysfunction are increasingly well understood. For acute acoustic trauma, Liberman and Kujawa [8] and Wang et al. [13] have described cochlear synaptopathy permanent damage to the synapses between inner hair cells and auditory nerve fibres that can occur at exposure levels that do not produce permanent threshold shifts. This condition, often referred to as “hidden hearing loss,” may explain why some individuals with a history of acoustic trauma report difficulty understanding speech in noisy environments despite having normal pure-tone audiometry [8,13,14]. Shenoy et al. [14] identified auditory brainstem response (ABR) as the most common diagnostic method for assessing cochlear synaptopathy, with reduced wave I amplitude serving as a potential electrophysiological marker.

For blast injury, Patterson and Hamernik [9] demonstrated that the rapid overpressure wave generates a combination of mechanical disruption, inflammatory responses, and apoptotic pathways that affect multiple structures of the auditory system. Cave et al. [1] reported that mixed deafness was the most common finding following blast injury, with vertigo present in a substantial proportion of cases. Fausti et al. [16] emphasised that blast-related auditory dysfunction frequently involves both peripheral and central components, requiring comprehensive evaluation and management.

In TBI, Gallun et al. [7] and Oleksiak et al. [6] provided evidence that diffuse axonal injury can disrupt central auditory processing networks, leading to deficits in temporal processing, sound localisation, and speech perception in noise. These deficits may persist even after peripheral hearing thresholds have returned to normal, underscoring the need for specialised central auditory testing in TBI populations.

### 9.4. Clinical Implications

The findings of this review have several important implications for clinical practice.

The high number of cases of auditory dysfunction following blast injury and acute acoustic trauma supports the implementation of routine audiological screening in at-risk populations, including military personnel, first responders, and individuals exposed to industrial or recreational noise hazards. Saunders and Griest [15] emphasised the need for hearing conservation programmes, including education, hearing protection, and regular audiometric monitoring, to reduce the burden of noise-induced auditory injury.

Recognition that early treatment is associated with improved recovery outcomes, as documented in clinical guidelines for sudden sensorineural hearing loss [10], supports the prompt evaluation and management of trauma-related auditory dysfunction. Chandrasekhar et al. [10] recommended the administration of corticosteroids within 72 h to two weeks of onset to maximise the likelihood of hearing recovery. Tripathi and Deshmukh [11] and Tardim Lopes et al. [12] provided additional evidence for the efficacy of both systemic and intratympanic corticosteroid therapy, with intratympanic administration serving as a salvage option when conventional therapy fails.

Identification of central auditory processing deficits in TBI patients with normal audiograms suggests that standard audiological evaluation alone may be insufficient. Clinicians should consider referral for specialised central auditory processing assessment in TBI patients who report auditory difficulties, particularly difficulty understanding speech in noisy environments, even when pure-tone thresholds are normal [5,6,7].

Concept of hidden hearing loss due to cochlear synaptopathy [8,13,14] has important implications for medicolegal evaluation and disability determination. Individuals with a history of acoustic trauma may have permanent auditory injury that affects real-world communication abilities despite normal audiometric thresholds. This finding highlights the need for objective electrophysiological measures, such as ABR, in the assessment of such patients [14].

Substantial burden of auditory dysfunction among military personnel, as documented by Swan et al. [17] and Fausti et al. [16], underscores the importance of preventive measures, early detection, and long-term rehabilitation services for veterans. Hearing conservation programmes, pre- and post-deployment audiometric monitoring, and access to audiological rehabilitation should be standard components of military healthcare.

### 9.5. Implications for Research

The findings of this review identify several priorities for future research.

Prospective longitudinal studies with standardised assessment protocols are needed to better characterise the natural history of trauma-associated tinnitus and hearing loss. Such studies should include comprehensive audiological evaluation (pure-tone audiometry, speech perception testing, ABR, and central auditory processing assessment) at baseline and at regular intervals following injury [5,8,13,14].

Further investigation of cochlear synaptopathy and hidden hearing loss is required to develop reliable diagnostic criteria and effective treatments. Wang et al. [13] and Shenoy et al. [14] noted that ABR remains the most common diagnostic method, but standardisation of protocols and normative data are needed. Additionally, research into neuroprotective agents or regenerative therapies that could prevent or reverse synaptic damage is a priority.

Randomised controlled trials of treatment protocols for trauma-related hearing loss are needed. While guidelines for idiopathic sudden sensorineural hearing loss exist [10], the evidence base for corticosteroid therapy specifically in acoustic trauma or blast-related hearing loss remains limited. Questions regarding optimal timing, dose, route of administration, and duration of therapy remain unanswered [10,11,12].

Research on central auditory processing deficits following TBI should be expanded. Le et al. [5] and Gallun et al. [7] noted substantial variability in assessment methods across studies, limiting comparability. Standardised test batteries and normative data for central auditory processing are needed to improve diagnostic accuracy and guide rehabilitation.

Limited evidence on whiplash-associated auditory dysfunction represents a significant knowledge gap. Ferrari and Russell [28] called for further epidemiological and mechanistic studies to determine the incidence, pathophysiology, and clinical course of auditory symptoms following whiplash injury.

Psychosocial consequences of trauma-associated tinnitus and hearing loss deserve further attention. Kreuzer et al. [4] documented that trauma-associated tinnitus is often accompanied by psychological distress, including anxiety and depression. Baguley et al. [30] and Henry et al. [32] reviewed the impact of tinnitus on quality of life, sleep, concentration, and social functioning. Dobie [18] discussed the medicolegal aspects of hearing loss evaluation. Integrated models of care that address both audiological and psychological needs may improve outcomes for affected individuals.

### 9.6. Strengths of the Review

This narrative review has several strengths. First, it synthesises evidence across multiple trauma mechanisms (blast, acoustic trauma, TBI, head trauma, whiplash), providing a comprehensive overview of trauma-associated auditory dysfunction. Second, it includes both military and civilian populations, enhancing the generalisability of the findings. Third, it integrates pathophysiological mechanisms with clinical patterns, offering a framework for understanding the observed variability in different outcomes. Fourth, it identifies specific knowledge gaps and proposes actionable directions for future research.

### 9.7. Limitations of the Review

Several limitations should be acknowledged. First, as a narrative review, this study did not perform a formal meta-analysis or quantitative risk of bias assessment. The qualitative synthesis, while appropriate for the heterogeneous evidence base, is subject to the inherent subjectivity of the reviewers [19,20,21,22]. Second, substantial heterogeneity across studies in population characteristics, injury severity, diagnostic criteria, and follow-up duration limits the comparability of findings and precludes the calculation of pooled prevalence estimates. Third, the possibility of publication bias cannot be excluded; studies with positive or striking findings may be overrepresented in the literature [31]. Fourth, the restriction to English-language publications may have introduced language bias [31].

The following methodological limitations are acknowledged:

- **Selection bias**: Despite a comprehensive search, relevant studies may have been missed.
- **Qualitative synthesis**: No quantitative pooling of results.
- **Heterogeneity**: High variability across studies limits comparability.
- **Publication bias**: Studies with positive findings may be overrepresented.
- **Language restriction**: Only English-language publications were included.
- **Narrative synthesis**: Inherent subjectivity in thematic analysis.
- **No formal quality scoring**: Risk of bias is not quantified.

These limitations are typical for narrative reviews and do not invalidate the conclusions but should be considered when interpreting the findings.

### 9.8. Methodological Considerations

The methodological quality of the included studies varies considerably. Observational studies, case series, and systematic reviews each have inherent strengths and limitations. The Newcastle–Ottawa Scale is one tool that could be used to assess the quality of non-randomised studies in future systematic reviews, but it was not applied in this narrative review. The search strategy followed best practices, including the use of multiple databases, reference list screening, and forward citation tracking [23,24,25,26,27]. However, the absence of a formal quality assessment limits the ability to weight findings by study quality.

### 9.9. Future Directions

Based on the findings of this review, the following future directions are recommended:

- **Standardisation of assessment protocols**: Adoption of uniform diagnostic criteria and test batteries for tinnitus and hearing loss following trauma would facilitate cross-study comparison and meta-analysis.
- **Prospective cohort studies**: Longitudinal follow-up of trauma-exposed individuals with serial audiological assessments would clarify recovery trajectories and prognostic factors.
- **Randomised controlled trials**: High-quality trials of corticosteroid therapy and other interventions for trauma-related hearing loss are needed to establish evidence-based treatment protocols.
- **Research on hidden hearing loss**: Further investigation of cochlear synaptopathy, including diagnostic methods and potential treatments, is a priority [8,13,14].
- **Central auditory processing in TBI**: Studies examining the prevalence, natural history, and rehabilitation of central auditory deficits following TBI are needed [5,6,7].
- **Whiplash and auditory dysfunction**: Epidemiological studies to determine the incidence and mechanisms of auditory symptoms following whiplash are required [28].
- **Psychosocial interventions**: Development and evaluation of integrated audiological and psychological interventions for trauma-associated tinnitus [4,30,32].
- **Prevention**: Research on the effectiveness of hearing protection devices and blast mitigation strategies in military and occupational settings should continue [15,17].

## 10. Conclusions

The available evidence consistently demonstrates that trauma-associated tinnitus and hearing loss are frequent and clinically significant consequences of blast injury, acute acoustic trauma, TBI, head trauma, and whiplash. Distinct pathophysiological mechanisms produce different patterns of auditory dysfunction, with blast injury commonly causing mixed hearing loss, acoustic trauma causing sensorineural loss with possible hidden hearing loss, and TBI causing central auditory processing deficits.

Recovery is variable and often incomplete, and early treatment is associated with better outcomes. The concept of hidden hearing loss due to cochlear synaptopathy has important implications for diagnosis and medicolegal assessment. Future research should focus on standardised assessment protocols, prospective longitudinal studies, randomised controlled trials of treatments, and investigation of central auditory processing deficits and whiplash-associated auditory symptoms.

## Figures and Tables

**Table 1:** Distribution of studies by trauma type.

Trauma Type	Frequency in the Literature	Representative Studies
Blast injury	Very frequently studied	Remenschneider et al., 2014 [2]; Patterson & Hamernik, 1997 [9]
Acute acoustic trauma	Very frequently studied	Ryan et al., 2016 [3]; Liberman & Kujawa, 2017 [8]
Traumatic brain injury (TBI)	Frequently studied	Oleksiak et al., 2012 [6]; Gallun et al., 2012 [7]
Head trauma (without documented TBI)	Less frequently studied	Included in systematic reviews
Whiplash-associated disorders	Rarely studied	Ferrari & Russell, 1999 [28]

**Table 2:** Reported clinical patterns by trauma type.

Trauma Type	Tinnitus	Hearing Loss	Predominant Hearing Loss Type
Blast injury	Very frequently reported	Very frequently reported	Mixed (conductive + sensorineural) [2,12]
Acute acoustic trauma	Very frequently reported	Very frequently reported	Sensorineural, often with 4 kHz notch [6,9]
Traumatic brain injury (TBI)	Frequently reported	Frequently reported	Sensorineural or central auditory processing deficits [20,23]
Head trauma	Reported	Reported	Variable; may be conductive or sensorineural
Whiplash	Reported but limited evidence	Reported but limited evidence	Not well characterised [24]

**Table 3:** Reported types of hearing loss following trauma.

Hearing Loss Type	Commonly Associated Trauma Mechanisms	Key Features
Sensorineural (SNHL)	Acoustic trauma, blast, TBI	High-frequency loss, 4 kHz notch [6,9]
Conductive (CHL)	Blast, head trauma	Tympanic membrane perforation, ossicular disruption [2]
Mixed (MHL)	Blast	Combination of conductive and sensorineural components [12]
Central auditory processing deficits	TBI	Difficulty understanding speech in noise despite normal audiometry [20,23]

**Table 4:** Reported recovery patterns by trauma type.

Trauma Type	Reported Recovery Patterns
Blast injury	Variable; tympanic membrane perforations often heal spontaneously; sensorineural component frequently persists [2,12]
Acute acoustic trauma	May show partial recovery; high-frequency notches often permanent [6,9]
Traumatic brain injury (TBI)	Central auditory deficits may persist even when peripheral thresholds recover [20,23]
Head trauma	Recovery depends on specific structural injury (e.g., tympanic membrane, ossicles)
Whiplash	Limited evidence; symptoms may be transient or persistent [24]

**Table 5:** Reported prognostic factors for recovery following traumatic auditory injury.

Prognostic Factor	Reported Association
Timing of treatment	Early treatment (within days to 2 weeks) associated with better recovery [27]
Severity of initial hearing loss	More severe loss associated with poorer prognosis
Presence of vertigo	May indicate more extensive inner ear injury
Patient age	Younger age may be associated with better recovery
Tympanic membrane integrity	Perforation may heal spontaneously; persistent perforation may require surgery [2]

**Table 6:** Summary of pathophysiological mechanisms by trauma type.

Trauma Type	Primary Mechanisms	Key Structures Affected
Acute acoustic trauma	Mechanical disruption, metabolic exhaustion, cochlear synaptopathy	Outer hair cells (basal turn), synapses between inner hair cells and auditory nerve fibres [6,9,10]
Blast injury	Overpressure wave, barotrauma, secondary and tertiary blast effects	Tympanic membrane, ossicular chain, basilar membrane, cochlear hair cells, central auditory pathways [2,12,13]
Traumatic brain injury (TBI)	Diffuse axonal injury (microscopic damage to the nerve fibers that transmit auditory and balance information from the ear to the brain), acceleration–deceleration forces	Central auditory pathways (inferior colliculus, medial geniculate body, auditory cortex) [14,15,23]
Whiplash	Proposed: vertebrobasilar ischemia, sympathetic dysfunction	Uncertain; possibly inner ear or central pathways [24]

## Data Availability

The data presented in this study are available on request from the corresponding author.
